# Corruption and instutitions: An analysis for the Colombian case

**DOI:** 10.1016/j.heliyon.2020.e04874

**Published:** 2020-09-18

**Authors:** Nicolás Ronderos Pulido, Alexander Cotte Poveda, Jorge Enrique Martínez Carvajal

**Affiliations:** a Universidad Santo Tomas, Carrera 9 No 51-11, Bogotá, Colombia; b Faculty of Economics, University Santo Tomas, Carrera 9 No 51-11, Bogotá, Colombia

**Keywords:** Corruption, Government, Binary choice, Economics, Behavioral economics, Economic development, Microeconomics, Econometrics

## Abstract

This paper identifies the main determinants of errors in the allocation of spending by the Colombian Government. Using information from the Electronic Public Procurement System (SECOP), the determinants of the probability of an addition to a contract are identified. The errors of the government can be interpreted as an approximation of their corruption. The average income and educational level of a colombian department are found to directly influence the probability of an addition. Using the estimation of the binary choice models, the forecast error of an addition is estimated, it is found that public and civil works contracts have more forecast error, forming an ideal mechanism for thefts and accumulation of bribes. Our results show that predicting an addition can be done with high certainty.

## 1 Introduction

It is generally accepted that public corruption implies a misallocation of state resources and a deterioration of the welfare of the members of a country (Dimant and Tosato, 2018). With corruption, public resources are assigned based on the search for lucrative activities, (see Kurer, 1993; Bhagwati, 1982; Krueger, 1974; Goel and Nelson, 2010, Berdiev et al., 2020 and Colonnelli and Prem, 2020). In countries with high levels of corruption, entrepreneurs are aware that they will have to pay bribes for permits and licenses; corruption is a tax that reduces levels of investment, capital accumulation and economic growth (Dell’Anno and Teobaldelli, 2015 and Gründler and Potrafke, 2019). Likewise, if corruption is highly profitable, people with great abilities could self-select to work in corrupt activities, decreasing the aggregate levels of education and technological progress (Treisman, 2007).

Now, historically, in Colombia, innumerable cases of corruption have been observed. The best-known cases have been presented in the public sector and are related to civil works and drug trafficking. Some of them are publicly known, others the evidence is scarce or altered, Hoggard (2004). In terms of perception of corruption, Colombia is ranked around the 50th percentile in the world, De Maria, W. (2008). Corruption has already been studied in Colombia, Langbein and Sanabria (2013) study bribery requests and find that corruption is a stable but variant phenomenon within the country. So Cotte (2015) studies the interactions between insecurity and corruption, it is found that insecurity and corruption have different trends, however the departments with a higher level of corruption and insecurity show less economic growth. In this paper, a measurement of corruption is proposed using a new database, with quantitative information, totally ruling out the perception of corruption. Our results allow us to better understand the behavior of the Government under activities highly related to corruption.

In the literature there are proposals to reduce levels of corruption. For example Rose-Ackerman (1975) formulates a model in which the government can competitively allocate tenders for public contracts or assign them according to a bilateral monopoly. It is found that crime can be reduced by modifying the contractual terms and the market structure. On the other hand lack of transparency, consistency, responsibility of the Governors, weakness of the judicial and legislative systems are classified as the main causes of corruption, Myint (2000) and Barrett and Fazekas (2020).

In quantitative terms, corruption studies are scarce, because there are no unique measures of corruption, and they are usually based on perception surveys. However, the correlation between different corruption indices is high, so there is a consensus in terms of the definition of corruption, Mauro (1995), Gutmann et al. (2020) and Ojeka et al. (2019). The aggregate effect of corruption on economic growth has been studied in the literature. For example, trade restrictions generate import and export permits to increase in value, generating incentives to offer and receive bribes, Ades and Di Tella (1999) find the magnitude of openness of an economy is inversely related to its levels of corruption.

If corruption is defined as the magnitude of tax evasion, the growth of the underground economy can be interpreted as greater corruption, affecting the level of public spending. Corrupt Governors generally prefer to spend public spending on activities that allow them to secretly accumulate bribes. Government personnel who want to steal public resources are more likely to spend a large part of their spending on goods and services that are difficult to value, that occur in highly concentrated sectors of the economy, and in large war or infrastructure projects, (Shleifer and Vishny, 1993 and Mohamedbhai (2020)). Therefore, a corrupt government is less likely to promote activities that generate difficulties in stealing public resources, such as: spending on public education, infrastructure to protect the environment, subsidies for old age, among others, Mauro (1995).

At a more disaggregated level, there are also studies on the causes of corruption. For example, when public employees have low wages relative to private employees, according to efficiency wages, government employees will have incentives to accept bribes (see Haque and Sahay, 1996 and Kraay and Van Rijckeghem, 1995). Thus, the education and culture of a society can influence levels of corruption, for example in countries with a wide variety of ethnic groups it is more likely to have more disorganized and corrupt systems, Shleifer and Vishny (1993). Similarly, public servants are more likely to offer favors to friends and family in societies where relationships are more personalized, Tanzi (1994).

More recently, Dimant and Tosato (2018) perform a literature review on the main determinants and effects of corruption. They find the expected effects of the interdependence between corruption and: i) bureaucracy, ii) economic and press freedom, iii) poverty, iv) wages and v) the growth of the underground economy (see Tanzi 1998, Goel and Nelson 2010, Treisman 2007, Paldam, 2002 and Owusu et al. (2019)). However, the most recent investigations give conflicting results on the interdependence between corruption and: i) competition of markets and politics, ii) foreign direct investment and iii) income inequality (see Goel and Nelson 2010, Dell'Anno and Teobaldelli 2015, Alexeev and Song 2013, Sharafutdinova, 2010 and Alfada (2019)).

The recent implementation of experimental econometric methods and different identification strategies have allowed the study of the relationships between corruption with gender, brain drain, and migration (Frank et al., 2011; Andersen et al., 2011; Dimant et al., 2015).

There is a branch of literature that seeks to measure the effects of corruption with objective data, instead of using the perception of corruption. This work is in line with this literature, which seeks to explain objective measurements of errors in the allocation of government resources. See for example Gorodnichenko and Peter (2007), Ferraz and Finan (2011), Chatterjee and Ray (2009) and Lima and Delen (2020) for a review of papers using micro data from local government audits, self-reports of bribery and crime.

The main contribution of this work is to use information from the Government itself to estimate the likelihood of corruption in the allocation of Government resources. In this paper, the main drivers of errors in the allocation of government resources for public expenditure are established. Results indicate that contract additions may result from poor forecasting or corruption of public officials. It is found that increases in the average income generate increases in the additions to public contracts. It is found that the Departments with more educated individuals have a higher probability of additions. The difficulty of anticipating additions by type of contract is also analyzed. It is found that the contracts with the greatest forecasting difficulties are those with the most additions. Using the results possible cases of corruption can be detected. It is recommended to use this type of estimations, by type of contract, to determine whether or not a contract actually requires an addition.

This document is divided into six sections including this introduction. The second section describes the database. The methodology, of binary choice models, is described in the third section. In the fourth section the main determinants of contract additions are identified. In the fifth section, a model is designed to forecast additions. Finally, some conclusions and recommendations are made.

## 2 Dataset

**Figure 1:**
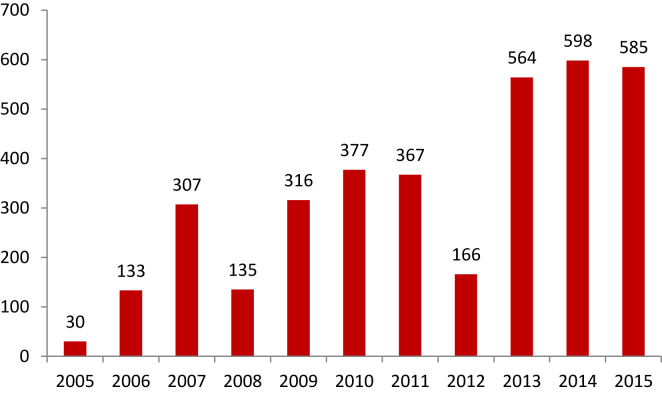
Number of contracts per year.

Source: SECOP II and authors' construction

Estimates are calculated using the following sources of information: i) SECOP II, ii) DANE iii) and the Labor Observatory. The Electronic Public Procurement System (SECOP II) has information on the monitoring of the execution of the contracts established between the Government and private entities. SECOP data was obtained at the departmental level of 4,463 contracts executed from 2005 to 2015. Figure 1 shows the number of contracts per year.

**Figure 2:**
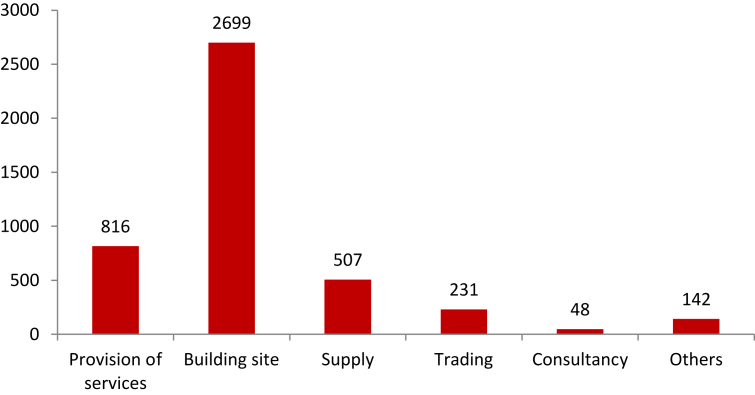
Type of contract.

Source: SECOP II and authors' construction

Likewise, there is information on the destination of the contracts. Historically, the largest number of government contracts occurs for the execution of public and civil works, followed by the provision of services and the supply of goods and services. In the last category are manufacturing, technology and communications, and commercial vehicles. Figure 2 shows the number of contracts and their typology.

**Figure 3:**
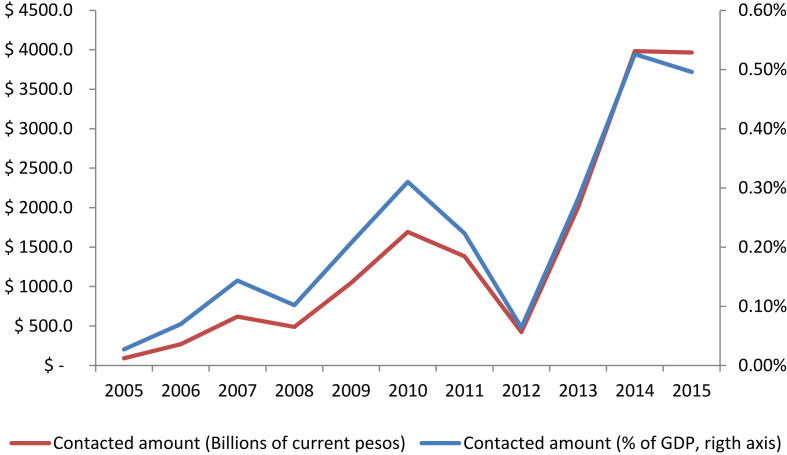
Contracted value.

Source: SECOP II and authors' construction

In addition from SECOP the monetary value of each of the contracts obtained. Figure 3 shows this value expressed in billions of current Colombian pesos and as a percentage of GDP. It can be seen that on average the value of public contracts amounts to 0.2% of GDP. Between 2005 and 2010, a constant growth of the contracted value can be observed, while from 2011 to 2015, a break can be observed, this can be attributed to the changes in spending priorities established by the current administration. SECOP II also uses information on additions to agreed contracts, the status of contracts, and the number of bidders in tenders.

From DANE, at the departmental level the following information was obtained: GDP per capita, the percentage of the population with unsatisfied basic needs (UBN), and from the vital statistics the number of homicides and the population are calculated. From the labor observatory, information on the number of graduates in higher education at the departmental level was obtained. The variables in the following table are used in the estimations (see Table 1).

**Table 1:** Variables definitions.

Variables	Description	Source
GDP_per	GDP per capita in millions of pesos	DANE
Graduates	Thousands of graduates in higher education	Labor observatory
Civil Work	Civil work contract	SECOP II
Provision of services	Provision of services contract	SECOP II
Supply	Supply contract	SECOP II
Homicides	Thousands of homicides	DANE
UBN	Unsatisfied basic needs index	DANE
Contract value	Contract value in billions of pesos	SECOP II
Proponents	Number of bidders in tenders	SECOP II

## 3 Methodology

This section explains the estimation of binary choice models. This method is adopted since our dependent variable is the probability of an event of corruption. The aim is to explain the probability of an addition to a conditional contract to a set of variables.

(1) Prob(Addition ∥X)=F(XB)

**Figure 4:**
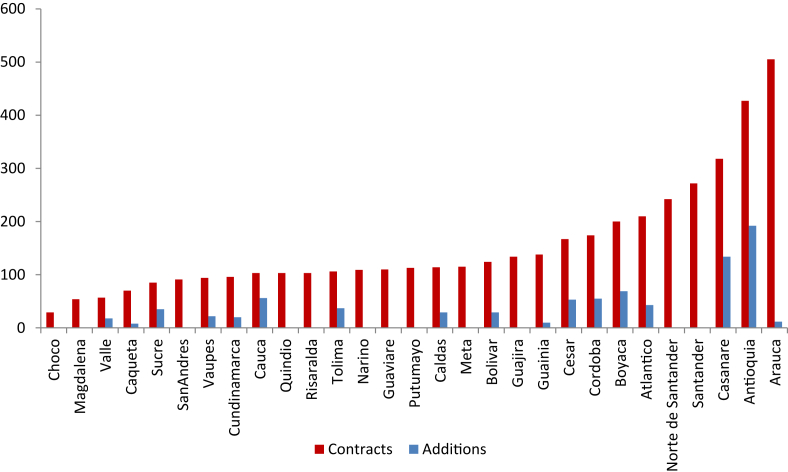
Number of contracts and additions by department.

Source: SECOP II and authors' construction

In this type of binary choice events, the Government selects the option that generates the most utility. By observing the event, an inference can be made about the utility of the government when granting an addition to a contract. Thus, the government finds a benefit by prioritizing some departments over others. This government decision depends on its utility function and that of its policy makers. In the same way, the departments with few additions will present few contracts, so that the presence of the government in those departments will be scarce. Figure 4 shows the number of contracts and additions by department, it can be seen that, on average, the departments with little (great) presence of the state have few (many) additions.

Likewise, additions to a project may occur due to errors in the evaluation and planning of the allocation of resources for a project. It is common to observe that the additions occur in the form of a higher budget than the one initially agreed or with extensions to the delivery times.

The functional form of the functionF(XB) defines the type of discrete choice model; it is usually assumed that this is a cumulative distribution function. For our estimation we use, i) a linear probability model and ii) a logit model. In the case of the linear probability modelF(XB) takes the form of:

(2) F(XB)=XB

For the first function, the estimation of the coefficients is carried out by GLS, weighted by the adjusted values of the model estimated by OLS and by the total departmental population. This estimator is used since OLS presents heterocedasticity, generating inefficiency. To demonstrate this, the error term when the event occurs (P=1) with probability XB isU=1−XB, and when does not occur (P=0) with probability1−XB, the error is U=−XB. Therefore, the expected value of the error conditional on the independent variables is given by E(U|X)=(1−XB)XB+(−XB)(1−XB)=0, thus the variance of the error $Var\left(U|X\right)={\left(1-XB\right)}^{2}XB+{\left(-XB\right)}^{2}\left(1-XB\right)=XB\left(1-XB\right)$ i.e. the variance of the error depends on the value of the independent variables. Therefore the GLS estimator of the form $B^{GLS}={\left(X^{\prime }{\text{Ω}}^{-1}X\right)}^{-1}X^{\prime }{\text{Ω}}^{-1}Y$ satisfies Gauss-Markov theorem, where each element of the diagonal of ${\text{Ω}}^{-1}$corresponds to $\frac{1}{\sqrt{\hat{p}\left(1-\hat{p}\right)}}$.

In the case of the logit model, the $F\left(XB\right)=\text{Λ}\left(XB\right)=\frac{e^{XB}}{1+e^{XB}}$ logistic function is used. This allows, unlike the linear probability model, to limit the model's predicted probabilities to the interval$\;\hat{p}\in \left[0,1\right]$. This advantage conditions a nonlinear relation of the independent variables with the dependent. The logit model estimator is calculated by maximum likelihood. The following log likelihood function is maximized.

(3) $$L\left(y_{1},\ldots ,\;y_{n}\right)=\overset{n}{\underset{i=1}{\sum }}y_{i}\;\ln\left(F\left(XB\right)\right)+y_{i}\text{ln}\left(1-F\left(XB\right)\right)$$

Using the logit estimates, the marginal effects are calculated, these determine the change in the probability of addition as a consequence of a change in the independent ones. Marginal effects are estimated as:

(4) $$\frac{dProb\left(\text{Addition }∥X\right)}{dX}=\text{Λ}\left(XB\right)\left(1-\text{Λ}\left(XB\right)\right)B$$

And their standard errors are calculated using the delta method. See Greene (2003).

## 4 Causes of contracts additions

Table 2 shows the estimates of the linear probability model. It can be observed that increases in the average income of a department are associated with a higher probability of additions. The Government usually prioritizes in its spending agenda the main cities of the country, since there are highly productive agglomerations. Incurring errors in the allocation of value and time of the contract involves large costs for the government and general welfare, costs can be reduced by making additions to the contracts. Now extensions to contracts can also be associated with corruption of those involved, where an extension in the deadline or value generates greater opportunities for theft of public resources. In this way, the departments with the highest average income show more thefts. In magnitude, the effect of one million pesos more (330 USD) in the average income generates an increase of 0.1% in the probability of additions.

**Table 2:** Linear probability model.

Variables	(1)	(2)	(3)	(4)	(5)	(6)
	additions	additions	additions	additions	additions	additions
GDP_per	0.0371∗∗∗	0.00571∗∗∗	0.00534∗∗∗	0.00618∗∗∗		0.00824∗∗∗
	(0.00193)	(0.00158)	(0.00157)	(0.00151)		(0.00211)
Graduates		0.000971∗∗∗	0.000969∗∗∗	0.000916∗∗∗		0.000849∗∗∗
		(4.68e-05)	(4.69e-05)	(0.000103)		(0.000109)
Civil Work			0.0167∗∗∗	0.0167∗∗∗	0.0183∗∗	0.0154∗∗
			(0.00619)	(0.00619)	(0.00734)	(0.00717)
Provision of services			0.00511	0.00507	0.00358	0.00398
			(0.0105)	(0.0105)	(0.0119)	(0.0119)
Supply			-0.00632	-0.00662	-0.0198∗	-0.00967
			(0.00844)	(0.00842)	(0.0102)	(0.00999)
Homicides				0.00842	0.124∗∗∗	0.0253
				(0.0152)	(0.00891)	(0.0163)
NBI					-0.00100∗∗∗	0.00123∗∗∗
					(0.000268)	(0.000287)
Contract value					-6.59e-05	-0.000342∗
					(0.000219)	(0.000206)
Proponents					0.000717∗	0.000389
					(0.000398)	(0.000275)
Constant	-0.143∗∗∗	-0.0683∗∗∗	-0.0724∗∗∗	-0.0815∗∗∗	0.0979∗∗∗	-0.153∗∗∗
	(0.0248)	(0.0180)	(0.0185)	(0.0181)	(0.0272)	(0.0359)
Observations	4,463	4,463	4,463	4,463	4,263	4,263
R-squared	0.269	0.373	0.375	0.375	0.312	0.382

Robust standard errors in parentheses ∗∗∗p < 0.01, ∗∗p < 0.05, ∗p < 0.1. It was controlled by 32 department dummy variables and 12 dummy variables of the year.

On the other hand, increases in education are associated with a higher probability of additions. This can be explained because of two effects i) the most educated people are aware of the objective of the contracts, so when the contract is breached they look for legal or social mechanisms to generate additions and ii) if those in charge of public procurement seek to steal resources, increases in their education could hide the thefts through additions to contracts. In magnitude an increase of one thousand graduates generates an increase in the probability of additions of 0.08%, on average in each department there is one hundred thousand graduates students per year.

The effect of homicides on the probability of additions is not significant when controlled by the average income and by the number of graduates. When we do not control for this variables an increase of one thousand homicides generates that the probability of additions increases 12.4%, historically in Colombia 500 people per year have been killed in each department, in 2009 there were about 400 thousand people killed in the country. This effect is explained because, on average, the departments with lower average income and fewer graduates are exposed to more violence. According to our estimates, the government does not determine the additions to the contracts according to the homicides. The estimation of the coefficients of the type of contract evidences a relevant result to curb corruption and improve efficiency in allocation of resources of the government, this is: the probability of additions increases substantially if the contract is civil works.

**Figure 5:**
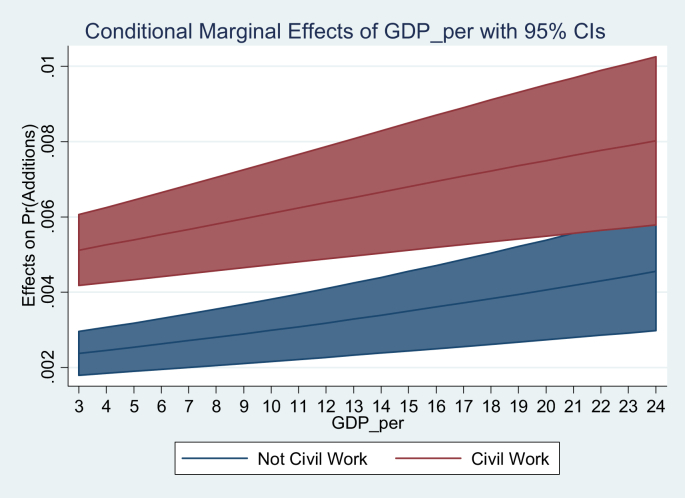
Marginal effects GDP per capita.

Table 3 shows the logit estimate. Although their coefficients cannot be interpreted to determine the magnitude, their sign establishes the qualitative effect on the probability of additions. It is well known that the logistic function is non-linear with respect to XB, so the influence of an independent on the probability of additions is nonlinear. Figure 5 shows the marginal effects of GDP per capita evaluated when: there is a public works contract, another contract category and the average characteristics of the sample.

**Table 3:** Logit estimate.

Variables	(1)	(2)	(3)	(4)	(5)	(6)
	additions	additions	additions	additions	additions	additions
GDP_per	0.0298∗∗∗	0.0292∗∗∗	0.0273∗∗∗	0.0289∗∗∗		0.0385∗∗∗
	(0.00417)	(0.00448)	(0.00454)	(0.00462)		(0.00475)
Graduates		0.00387∗∗∗	0.00401∗∗∗	0.00346∗∗∗		0.00463∗∗∗
		(0.000226)	(0.000233)	(0.000397)		(0.000446)
Civil Work			1.078∗∗∗	1.076∗∗∗	0.929∗∗∗	0.975∗∗∗
			(0.154)	(0.154)	(0.154)	(0.158)
Provision of services			0.832∗∗∗	0.820∗∗∗	0.638∗∗∗	0.761∗∗∗
			(0.184)	(0.184)	(0.182)	(0.188)
Supply			0.327	0.288	0.0878	0.353∗
			(0.203)	(0.204)	(0.205)	(0.209)
Homicides				0.102∗	0.596∗∗∗	0.160∗∗
				(0.0599)	(0.0408)	(0.0623)
NBI					0.0141∗∗∗	0.0323∗∗∗
					(0.00285)	(0.00329)
Contract value					0.00260	0.00123
					(0.00189)	(0.00205)
Proponents					0.00436	0.00538
					(0.00351)	(0.00392)
Constant	-1.907∗∗∗	-2.370∗∗∗	-3.218∗∗∗	-3.235∗∗∗	-3.229∗∗∗	-5.006∗∗∗
	(0.0725)	(0.0840)	(0.167)	(0.167)	(0.213)	(0.270)
Observations	4,463	4,463	4,463	4,463	4,263	4,263

Robust standard errors in parentheses ∗∗∗p < 0.01, ∗∗p < 0.05, ∗p < 0.1. It was controlled by 32 department dummy variables and 12 dummy variables of the year.

**Figure 6:**
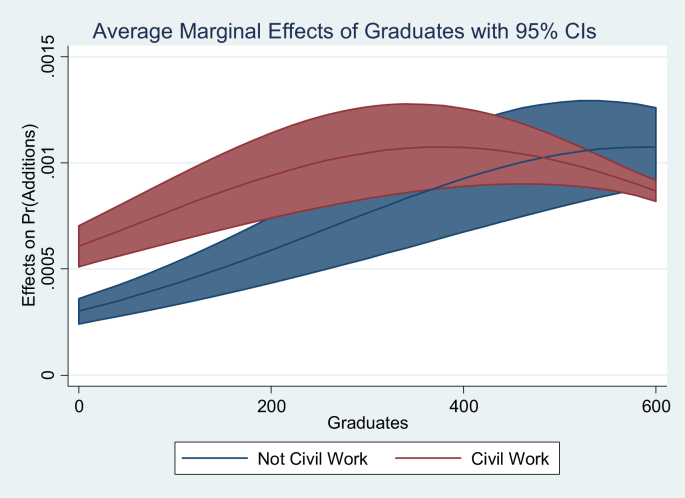
Marginal effects number of graduates.

If the contract is a public work, the effect of GDP per capita on the probability of additions is approximately double that for another type of contract. This can be explained due to the importance of public works for the general welfare, so these contracts must be completed by any means. Likewise, public works contracts present the ideal scenario for thefts, since on average the value of public works contracts represents 15% of all contracts. Figure 6 shows the estimation of the marginal effects of the number of graduates, they are evaluated in the average characteristics of the sample. A marginally decreasing and significant effect on the probability of additions is observed. The balance between the educated population that demands the government to fulfill the contracts, and the more educated Governors that modify the contract terms explains this result.

**Figure 7:**
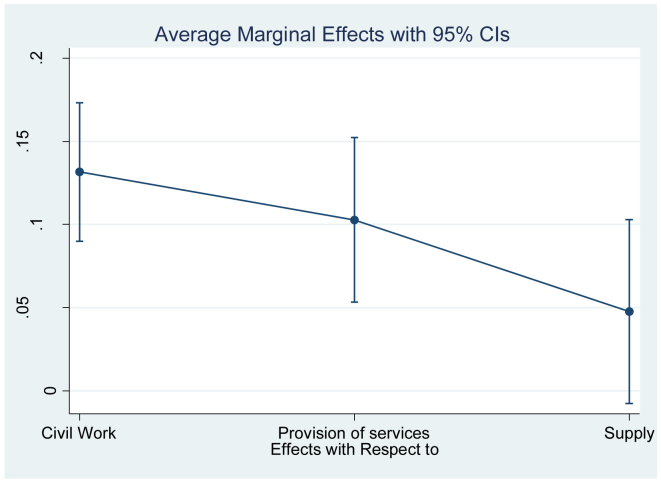
Marginal effects object of the contract.

Now Figure 7 shows the marginal effects of a change in the type of contract. The probability of addition is increased by 15% for civil works contracts relative to other types of contracts (purchase, lease, concession or consultancy). For the case of contracts of provision of services and supply the increase in the probability is 10% and 5%, respectively.

## 5 Forecasting contracts additions

The additions are generated due to non-compliance in the terms of the contracts and in the development of the projects. However, are these errors random? Or the government doesn't anticipate additions intentionally? To answer these questions, the predictive power of the additions is calculated using the estimates of the logit model. The forecast is estimated as:

(6) $$\begin{array}{cc} F=\hat{Addition} & if\frac{e^{X\hat{B}}}{1+e^{X\hat{B}}}\geq \delta \\ \hat{F=No\;Addition} & if\frac{e^{X\hat{B}}}{1+e^{X\hat{B}}}<\delta \end{array}$$

**Figure 8:**
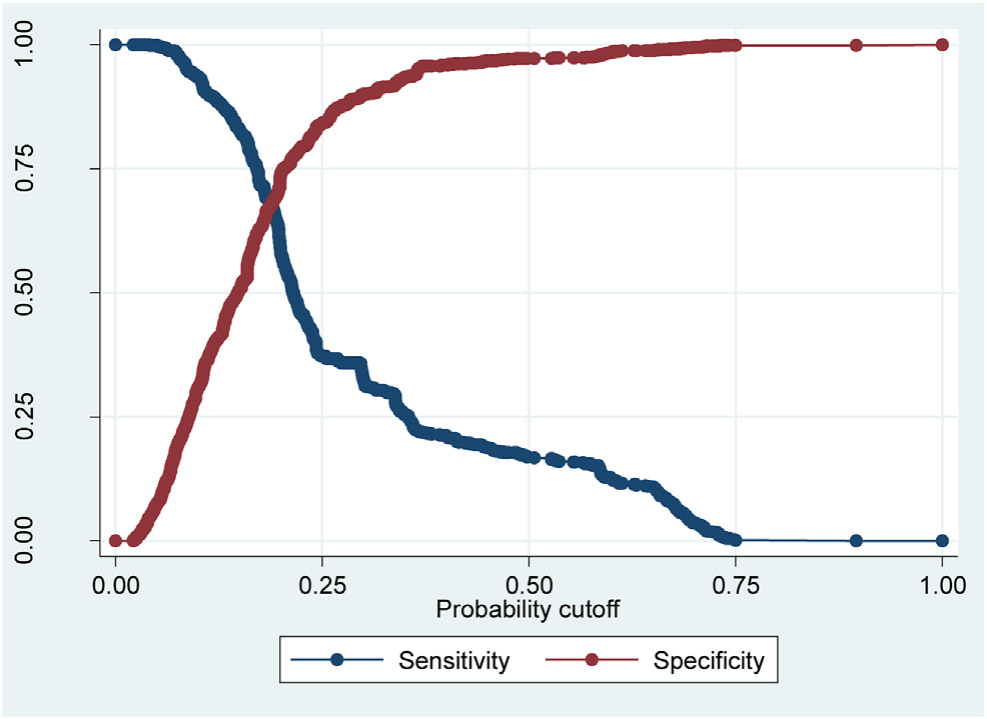
Selection of δ.

**Figure 9:**
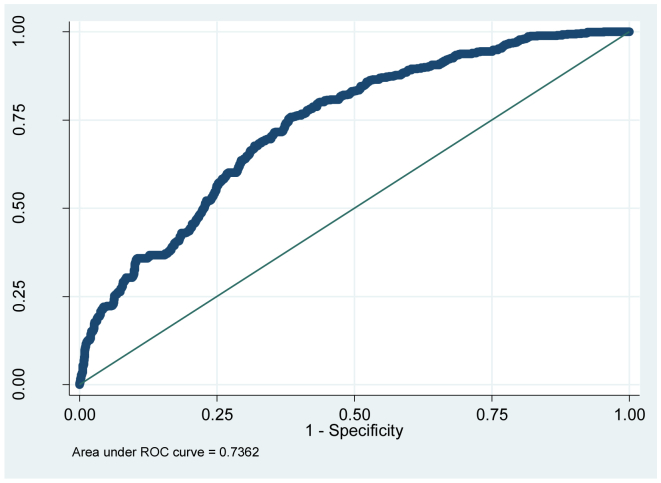
ROC curve.

Table 4 presents the forecast results and reports: i) the probability of classifying a contract as an addition conditional to it actually had additions, ii) the probability of classifying a contract with non-additions conditional on it actually had no additions and iii) the total percentage of correct classification. The parameter δ is the cut-off point, it is fixed maximizing the number of correctly classified contracts. Figure 8 presents the two probabilities, sensitivity and specificity, for all δ values and the entire sample. The value of δ in Figure 8 is 0.23. It can be observed that in supply contracts, civil works and other types of contracts, the prediction of additions implies a greater error, relative to service presentation contracts. However these measures are sensitive to the choice of δ. For this, predictive power is calculated using the area below the ROC curve, see Hardin and Hilbe (2012) for more details of estimating the ROC curve (see Figure 9).

**Table 4:** Area under the ROC curve.

	Civil work	Provision of services	Supply	Others	Total
Area under the ROC curve	0.7032	0.7068	0.7884	0.7610	0.7362
Hypothesis: $H_{o}:\mathrm{RO}C_{c_{i}}=\mathrm{RO}C_{\mathrm{cj}}$ (P-values)					
Civil work	0	-	-	-	-
Provision of services	97%	0	-	-	-
Supply	0	1.7%	0	-	-
Others	0	3.7%	34%	0	-
Total	2%	26%	12%	41%	0

The ROC curve statistics are estimated with standard bootstrap errors.

The ROC curve is a graph of sensitivity versus 1-specificity. Figure 9 shows the estimation of the curve. In a binary choice model with no predictive power the probability of classifying additions correctly is equal to the probability of classifying additions incorrectly, so the ROC curve will be a 45° line. The area below the curve is calculated using trapezoids, similar to the Gini coefficient. In a model with zero forecasting power the area under the curve will be 0.5, and with perfect forecasting power the area under the curve will be 1. For more details of the ROC curve see McClish (1989) and Kumar and Indrayan (2011).

Table 4 shows the results of the area under the curve for each type of contract. The hypothesis of whether the predictive power of the model is statistically equal for each type of contract (outside the main diagonal of the table), equal for all contracts (total on the table) and whether the area is statistically equal to 0.5 (on the diagonal of the table, zero predictive power) is also showed.

The forecasting power of the model depends on the type of contract. Civil works contracts present the greatest uncertainty when formulating the terms of the contracts, since the model applied to civil work contracts presents the least forecast power. Likewise for any type of contract, the model predicts the additions statistically better than the toss of a coin; in fact it can be predicted with great certainty when the area under ROC is above 0.7, McClish (1989). It can also be seen that the contracts with the least forecasting power have the highest probability of additions. All types of contracts, with the exception of civil works, have a forecasting power statistically equal to the total number of contracts. In the Colombian public media, corruption cases involving government contracts usually involve civil works. If those in charge of this type of contract seek to steal public resources; it is rational to use the most uncertain contracts, where additions can be justified due to poor planning.

## 6 Conclusions and recommendations

There is a recent branch of the corruption literature that seeks to explain corruption using micro data with observable variables that is, totally excluding perception. The main contribution of this paper is to identify a new measure to detect the possibility of corruption. It is built with observable information, without the need to use perception of corruption.

In this work, the main drivers of errors in the allocation of government resources for public expenditure are established. Contract additions may result from poor forecasting or corruption of public officials. It is found that increases in the average income generate increases in the additions to public contracts; the government concentrates on the contracts of the Departments with the highest income, due to costs in the general welfare and the search for theft opportunities. It is found that the Departments with more educated individuals have a higher probability of additions, as a consequence of i) more individuals who demand the fulfillment of their fundamental rights and ii) more educated corrupt rulers.

The difficulty of anticipating additions by type of contract is also analyzed. It is found that the contracts with the greatest forecasting difficulties are those with the most additions. The estimated model in this article allows us to forecast with great certainty whether or not a public contract will present an addition, even before it is presented. Public officials seeking to steal resources or accumulate bribes find it optimal to hide their behavior in contracts with forecasting difficulties and easy justification for additions. If the initial terms of public contracts are sufficient to fulfill the contractual objective, the additions would not be necessary and the mechanisms to steal and accumulate bribes would be mitigated. Additions should be made only in extraordinary cases.

In this way, using the results and binary choice estimates in this article, possible cases of corruption can be detected by generating additions above expectations. It is recommended to use this type of estimations, by type of contract, to determine whether or not a contract actually requires an addition.

## Declarations

### Author contribution statement

N. Ronderos: Conceived and designed the experiments; Performed the experiments; Analyzed and interpreted the data; Contributed reagents, materials, analysis tools or data; Wrote the paper.

A.C. Poveda: Analyzed and interpreted the data; Contributed reagents, materials, analysis tools or data.

J.E.M. Carvajal: Analyzed and interpreted the data; Contributed reagents, materials, analysis tools or data; Wrote the paper.

### Funding statement

This work was supported by Universidad Santo Tomas.

### Competing interest statement

The authors declare no conflict of interest.

### Additional information

No additional information is available for this paper.

## Appendix A Supplementary data

BaseOtos.dta
